# Seven days of warm‐water immersion enhances resting irisin and BDNF, but not klotho, in older men

**DOI:** 10.1113/EP093317

**Published:** 2026-03-04

**Authors:** Joel M Garrett, James J McCormick, Kelli E King, Kristina‐Marie T Janetos, Nathalie V Kirby, Fergus K O'Connor, Glen P Kenny

**Affiliations:** ^1^ School of Allied Health, Sport and Social Work Griffith University Queensland Australia; ^2^ Australian Centre for Precision Health and Technology (PRECISE) Griffith University Gold Coast Queensland Australia; ^3^ Human and Environmental Physiology Research Unit, School of Human Kinetics University of Ottawa Ottawa Ontario Canada; ^4^ Clinical Epidemiology Program Ottawa Hospital Research Institute Ottawa Ontario Canada

**Keywords:** ageing, climate change, heat stress, metabolic stress, neuroplasticity, oxidative stress, passive heat, thermoregulation

## Abstract

We examined whether seven consecutive days of warm‐water immersion could elevate resting and exercise‐induced levels of brain‐derived neurotrophic factor (BDNF), irisin and klotho in older adults. These biomarkers support cognitive and metabolic health, but their levels decline with age. Passive heat exposure, like warm‐water immersion, may offer a promising alternative to exercise for enhancing cellular‐level physiological resilience in populations where exercise is limited. Twelve habitually active older men (median [IQR] age: 68 [64–73] years; ${\dot{V}}_{O_{2}\mathrm{peak}}$: 34.1 [29.4–36.1] mL O_2_ · kg^−^^1^ · min^−^^1^) completed seven consecutive days of ∼90‐min warm‐water immersion (∼40°C), with rectal temperature maintained at ∼38.5°C for the final 60 min. Before and after the warm‐water immersion intervention, participants completed a standardized exercise–heat stress test consisting of three 30‐min cycling bouts (150, 200 and 250 W · m^−^^2^), each separated by 15‐min rest. Blood samples were collected at baseline and post‐exercise to assess circulating BDNF, irisin, and klotho. Repeated warm‐water immersion increased resting BDNF by 2.43 ng · mL^−1^ (95% CI: 0.64–4.23; *P* = 0.005; 1.73‐fold) and irisin by 0.94 ng · mL^−1^ (95% CI: 0.25–1.63; *P* = 0.005; 1.65‐fold). Resting klotho concentration did not change (mean difference: +0.05 ng · mL^−1^; 95% CI: –0.05 to 0.14; *P* = 0.539). No significant exercise‐induced changes in any cytoprotective hormones were observed (*P* ≥ 0.053). Seven consecutive days of warm‐water immersion elevated resting levels of BDNF and irisin, but not klotho, in older males. Passive heat acclimation may offer a non‐exercise strategy to upregulate biomarkers linked to cognitive and metabolic health, reflecting activation of cellular pathways associated with physiological resilience.

## Abstract (highlights)

- **What is the central question of this study?**
  Can 7 days of warm‐water immersion elevate resting and exercise‐induced concentrations of key biomarkers, BDNF, irisin, and klotho, in active older men?
- **What is the main finding and its importance?**
  Completing 7 days of warm‐water immersion increased resting levels of BDNF and irisin, but not klotho, in healthy, active older men. Exercise‐induced responses remained unchanged. These findings suggest heat acclimation can activate pathways associated with cellular resilience and metabolic regulation in this population and provide support for investigation of warm‐water immersion as a strategy for promoting resilience in older adults.

## 1 INTRODUCTION

Exercise is well known to support healthy ageing (Eckstrom et al., 2020) and enhance thermoregulatory function during heat stress (Stapleton et al., 2015), yet many older adults face barriers to regular physical activity (Kang et al., 2025). Consequently, alternative interventions capable of eliciting the cellular and systemic benefits of exercise without requiring physical exertion have increasingly garnered interest (Patrick & Johnson, 2021). Among these, passive heat therapy (e.g., warm‐water immersion, saunas) has emerged as a promising approach, demonstrating potential to improve cardiovascular health, endothelial function, and thermoregulatory efficiency, suggesting that thermal stress may induce adaptations comparable to those achieved through exercise‐based heat acclimation (Patrick & Johnson, 2021; Rodrigues et al., 2024). Such interventions may therefore enhance physiological resilience in ageing populations where exercise tolerance or access is limited.

Passive heat exposure induces mild hyperthermia that triggers a hormetic response facilitated by molecular mechanisms that mitigate protein damage and aggregation, as well as activate endogenous antioxidant, repair, and degradation processes (Patrick & Johnson, 2021). This response engages many of the same molecular pathways as exercise, including activation of heat shock proteins, oxidative stress defence, modulation of pro‐ and anti‐inflammatory factors, and mitochondrial biogenesis (Patrick & Johnson, 2021). Within this framework, certain circulating hormones act as biomarkers of these adaptive processes, reflecting activation of cytoprotective and stress‐regulatory signalling. Brain‐derived neurotrophic factor (BDNF) supports neuronal survival, synaptic plasticity, and neurogenesis (Lipsky & Marini, 2007; Marini et al., 2004), with higher levels associated with improved cognition and cardiovascular protection (Rothman et al., 2012). Repeated passive heat exposure elevates circulating BDNF in humans and increases hypothalamic BDNF expression in animal models, adaptations linked to improved thermoregulatory control and behavioural stress resilience (Chen et al., 2025; Glazachev et al., 2020). Irisin, a myokine cleaved from FNDC5, promotes metabolic adaptation and thermogenesis by enhancing adipose browning, insulin sensitivity, and antioxidant defences (Paoletti & Coccurello, 2024). Its release may be stimulated by heat stress as a protective response that limits oxidative stress and cellular damage (Wang et al., 2020). Additionally, irisin can cross the blood–brain barrier and upregulate hippocampal BDNF, supporting neuroprotection and cognitive function (Jodeiri Farshbaf & Alviña, 2021). Klotho, a renally derived anti‐ageing hormone, regulates multiple organ systems through its antioxidant and anti‐inflammatory properties and is typically reduced in states of chronic inflammation (Prud'homme et al., 2022). Given that ageing is accompanied by systemic inflammation and oxidative stress (Franceschi & Campisi, 2014), circulating klotho declines with age, making it a valuable biomarker of physiological resilience and a logical target for investigating heat‐induced cytoprotective adaptations. Together, BDNF, irisin, and klotho represent key mediators through which passive heat acclimation may mimic exercise‐induced adaptations, promoting cellular resilience, neuroprotection, and ultimately, healthy ageing.

Accordingly, this study aimed to determine whether a 7‐day warm‐water (∼40°C) immersion heat acclimation protocol alters resting and exercise‐induced concentrations of BDNF, irisin and klotho in habitually active older men, thereby assessing the potential of passive heat exposure to stimulate cellular pathways associated with physiological resilience. We specifically recruited healthy, aerobically fit older men to minimize inter‐individual variability and ensure the safety of prolonged passive heating and exercise testing under controlled laboratory conditions. This homogeneous sample was selected to establish proof‐of‐concept before extending to more diverse or clinically vulnerable populations. We hypothesized that in this group of older, fit men, passive heat acclimation would increase resting concentrations of BDNF, irisin and klotho and augment the acute hormonal response to exercise.

## 2 METHODS

This study was part of a larger investigation examining the effects of short‐term (7 days) passive warm‐water immersion on heat dissipation in older men (ClinicalTrials.gov: NCT05838612). The protocol was approved by the University of Ottawa Health Sciences and Science Research Ethics Board (H03‐21‐6731) and conducted in accordance with the *Declaration of Helsinki*. All participants provided written informed consent. Full methodological details are published elsewhere (Janetos et al., 2025). Key elements are summarized below.

### 2.1 Participants

Twelve healthy, habitually active men (60–80 years) participated. None were heat‐acclimated, defined as no environmental heat exposure in the preceding 3 weeks, nor engaged in occupations involving regular heat stress. Testing was conducted between April and June, and September and February to avoid seasonal acclimatization. The participants were instructed to refrain from exercise throughout the entirety of the study. Reported medications (e.g., statins, ACE inhibitors, antidepressants) were not expected to influence heat stress responses (Meade et al., 2020).

### 2.2 Experimental protocol

Participants underwent baseline screening, a pre‐acclimation exercise–heat stress test (Day 0; D0), seven consecutive days of warm‐water immersion (∼40°C), and a post‐acclimation exercise–heat stress test (Day 8; D8). Sessions were conducted at the same time of day to minimize circadian influence. Participants refrained from alcohol, caffeine, NSAIDs, and structured exercise for ≥24 h and consumed a light meal and 500 mL of water ∼2 h before testing.

### 2.3 Preliminary screening

As described in the first report of this series (Janetos et al., 2025), during the initial laboratory visit, participants underwent baseline assessments including body height, mass, surface area, and composition and peak aerobic capacity (${\dot{V}}_{O_{2}\mathrm{peak}}$).

### 2.4 Exercise–heat stress tests

Exercise–heat stress tests were completed the day before and after the 7‐day warm‐water immersion intervention using a modified Snellen direct air calorimeter, which allows for direct measurements of whole‐body heat exchange (Kenny & Jay, 2013). The calorimetry, metabolic and thermal measurement procedures are described comprehensively in Janetos et al. (2025). Participants rested in a thermoneutral room (∼23°C) for ∼30 min before entering the calorimeter (set to 40°C, ∼13% relative humidity). After 15 min of seated rest in the heat, participants performed three successive 30‐min bouts of semi‐recumbent cycling at target metabolic heat production rates of 150 W · m^−^^2^ (light; median [IQR]: 29 [16–31]% ${\dot{V}}_{O_{2}\mathrm{peak}}$), 200 W · m^−^^2^ (moderate; 42 [37–45]% ${\dot{V}}_{O_{2}\mathrm{peak}}$) and 250 W · m^−^^2^ (vigorous; 51 [44–57] % ${\dot{V}}_{O_{2}\mathrm{peak}}$), each separated by 15 min of recovery. These target heat production levels were selected based on prior calorimetry work showing that inter‐individual and age‐related differences in thermoregulatory function are most apparent at moderate‐to‐high metabolic heat loads (Kenny & Jay, 2013), thereby providing a sensitive test of the adaptive effect of short‐term passive heat acclimation.

### 2.5 Warm‐water immersion intervention

For each warm‐water immersion session, participants rested in a thermoneutral environment (∼22°C) for 30 min. Participants then entered a warm‐water bath maintained at ∼40°C immersed to the clavicle. Once a rectal temperature of 38.5°C was achieved, water temperature was adjusted as needed to maintain this core temperature for the remaining 60 min of immersion. This process was repeated each day of the 7‐day acclimation period.

### 2.6 Blood sampling and biochemical analysis

Venous blood was collected into K_2_EDTA BD Vacutainer tubes and serum separator tubes before and immediately after each exercise–heat stress test. Blood collected in serum separator tubes was allowed to coagulate for ∼15 min prior to centrifugation. Haematocrit and haemoglobin were quantified in duplicate (Ac T diff2; Beckman Coulter, Brea, CA, USA) and all serum protein concentrations were corrected for changes in plasma volume (Dill & Costill, 1974). The remaining blood was centrifuged (1380 × relative centrifugal force) for 10 min before plasma and serum were aliquoted and subsequently stored at –80°C until analysis.

Serum samples were assessed in duplicate using an enzyme‐linked immunosorbent assay (ELISA) for BDNF (Bio‐Techne, Minneapolis, MN, USA, cat. no. DY248), irisin (Bio‐Techne, cat. no. DY9420), and klotho (Bio‐Techne, cat. no. DY5334) according to the manufacturer's instructions. All proteins were diluted (1:5) in 1% bovine serum albumin prior to analysis and optical densities read on a microplate reader (Synergy, BioTek, Winooski, VA, USA) at 450 nm with a wavelength correction of 570 nm to minimize the influence of non‐specific wavelength emissions. The respective inter‐ and intra‐plate coefficient of variation for each assay were as follows: BDNF 2.88 and 5.11%, irisin 4.02 and 6.88%, and klotho 4.63 and 4.97%.

### 2.7 Statistical analysis

All analyses were conducted in R (version 4.4.0; 2024‐04‐24). Linear mixed‐effects models were used to examine fixed effects of day (D0 pre‐acclimation vs. D8 post‐acclimation), session (baseline [BS] vs. end‐of‐exercise [End‐Ex]), and their interaction (day × session) on biomarker concentrations. Participant ID was included as a random intercept. We used Type III Wald *F*‐tests to evaluate fixed effects, and estimated marginal means (EMM) with Tukey‐adjusted pairwise comparisons to test differences between all day × session combinations. Fold change was calculated as the ratio of EMMs to quantify relative magnitude and direction of change.

Model assumptions were assessed via Q–Q and residual‐versus‐fitted plots. Diagnostics indicated assumptions were adequately met for irisin and BDNF, but not klotho. Nonetheless, skewness (0.07–0.19) and kurtosis (0.06–2.47) values suggested approximately symmetric residuals with acceptable tails, supporting parametric robustness. Robust mixed‐effects models (Huber estimation) were also run for klotho to down‐weight outliers (Mohammadi & Kazemi, 2022). Results were consistent with standard models, supporting inference from raw values.

To test whether acute exercise‐induced changes (post‐exercise minus pre‐exercise) differed between time points, change scores were calculated separately for D0 and D8 and compared with a paired‐samples Student's *t*‐test. A two‐tailed α of *P* < 0.05 was considered significant.

## 3 RESULTS

### 3.1 Participant characteristics and baseline physiological responses

Full participant characteristics for individuals included within this study are presented in the first report of this series (Janetos et al., 2025); only details relevant to the current study are repeated herein for necessary context. Twelve habitually active men aged 60–80 years (mean weekly exercise: 153 min [range: 90–190] of moderate‐to‐vigorous intensity) participated in this study. Participant characteristics and baseline physiological measurements, assessed under thermoneutral conditions at rest before the exercise–heat stress tests on both D0 and D8, are presented in Table 1. Resting rectal temperature, mean skin temperature, and both systolic and diastolic blood pressure all decreased significantly after seven days of warm‐water immersion (all *P* ≤ 0.034), whereas resting heart rate remained unchanged (*P* = 0.242).

**TABLE 1:** Participant characteristics and resting baseline physiological responses adapted from Janetos et al. (2025).

Participant characteristics	All participants (*n* = 12) Median (IQR)
Age (years)	68 (64–73)
Height (m)	1.73 (1.70–1.79)
Body mass (kg)	73.4 (69.6–78.2)
Body mass index (kg · m^−2^)	24.6 (23.1–25.9)
Body surface area (m^2^)	1.86 (1.83–1.92)
Body fat (%)	20.6 (18.0–23.3)
${\dot{V}}_{O_{2}\mathrm{peak}}$ (mL O_2_ · kg^−1^ · min^−1^)	34.1 (29.4–36.1)

Baseline resting physiological responses	Pre‐acclimation (D0) Mean (SD)	Post‐acclimation (D8) Mean (SD)	Mean difference [95% CI]	*P*
Rectal temperature (°C)	36.9 (0.3)	36.6 (0.2)	−0.3 [–0.5, –0.1]	<0.001
Mean skin temperature (°C)	35.7 (0.4)	35.5 (0.7)	−0.2 [–0.5, 0.0]	0.034
Heart rate (beats · min^−1^)	68 (15)	66 (13)	−2 [–4, 1]	0.242
Systolic pressure (mmHg)	127 (11)	121 (10)	−6 [–11, –2]	0.012
Diastolic pressure (mmHg)	79 (10)	74 (6)	−5 [–9, 0]	0.033

Values for participant characteristics are the median and interquartile range (IQR). Values for baseline physiological response are mean and standard deviation (SD) or mean difference and 95% confidence interval [95% CI]. *P*‐value presented for a paired *t*‐test. Resting baseline physiological responses were taken during the instrumentation period in a thermoneutral environment before entering the direct air calorimeter.

### 3.2 Effect of passive heat acclimation on resting biomarker concentrations

**FIGURE 1:**
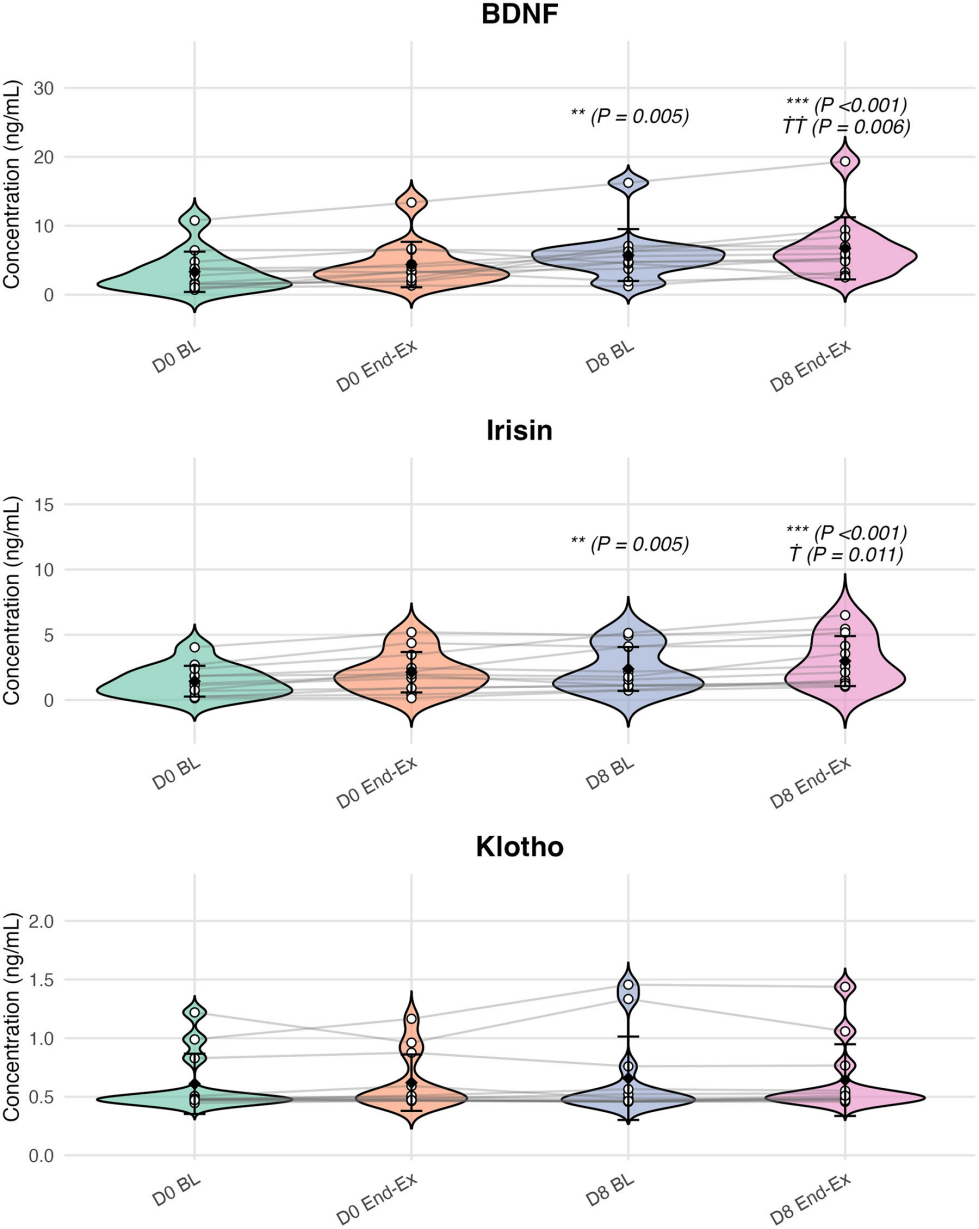
Concentrations of BDNF, irisin and klotho at four time points: D0 BL (baseline, pre‐exercise on Day 0), D0 End‐Ex (end of exercise on Day 0), D8 BL (baseline, pre‐exercise on Day 8), and D8 End‐Ex (end of exercise on Day 8). Individual participant values (*n* = 12) are shown in light grey, with paired time points connected by thin lines. Group means  standard deviation (SD) are overlaid as black diamonds. Symbols above each mean indicate significant pairwise differences relative to D0 BL (*) and D0 End‐Ex (†) time points.

Linear mixed‐effects modelling revealed large and statistically significant increases in resting concentrations of both BDNF and irisin following the 7‐day passive warm‐water immersion intervention (Table 2 and Figure 1). In contrast, klotho concentrations remained unchanged across time points (Table 2).

**TABLE 2:** Estimated marginal mean differences and fold changes in BDNF, irisin and klotho concentrations between baseline (BL) and end‐of‐exercise (End‐Ex) time points on Day 0 (D0) and Day 8 (D8).

Biomarker	Timepoint comparison	Reference time point	Comparison time point	Reference mean (SD)	Comparison mean (SD)	Mean diff [95% CI]	*P*	Fold change [95% CI]
BDNF	D0 BL vs. D0 End‐Ex	D0 BL	D0 End‐Ex	3.33 (2.92)	4.38 (3.30)	1.05 [–0.74, 2.85]	0.399	1.32 [0.60, 2.88]
BDNF	D0 BL vs. D8 BL	D0 BL	D8 BL	3.33 (2.92)	5.76 (3.76)	2.43 [0.64, 4.23]	0.005 **	1.73 [0.84, 3.56]
BDNF	D0 BL vs. D8 End‐Ex	D0 BL	D8 End‐Ex	3.33 (2.92)	6.73 (4.50)	3.40 [1.61, 5.20]	<0.001 ***	2.02 [1.01, 4.05]
BDNF	D8 BL vs. D8 End‐Ex	D8 BL	D8 End‐Ex	5.76 (3.76)	6.73 (4.50)	0.97 [–0.83, 2.76]	0.472	1.17 [0.73, 1.88]
Irisin	D0 BL vs. D0 End‐Ex	D0 BL	D0 End‐Ex	1.44 (1.18)	2.12 (1.55)	0.69 [–0.01, 1.38]	0.053	1.48 [0.69, 3.18]
Irisin	D0 BL vs. D8 BL	D0 BL	D8 BL	1.44 (1.18)	2.38 (1.68)	0.94 [0.25, 1.63]	0.005 **	1.65 [0.79, 3.46]
Irisin	D0 BL vs. D8 End‐Ex	D0 BL	D8 End‐Ex	1.44 (1.18)	2.98 (1.92)	1.55 [0.85, 2.24]	<0.001 ***	2.08 [1.03, 4.19]
Irisin	D8 BL vs. D8 End‐Ex	D8 BL	D8 End‐Ex	2.38 (1.68)	2.98 (1.92)	0.61 [–0.09, 1.30]	0.104	1.26 [0.77, 2.05]
Klotho	D0 BL vs. D0 End‐Ex	D0 BL	D0 End‐Ex	0.61 (0.26)	0.62 (0.24)	0.01 [–0.09, 0.11]	0.992	1.02 [0.69, 1.49]
Klotho	D0 BL vs. D8 BL	D0 BL	D8 BL	0.61 (0.26)	0.66 (0.36)	0.05 [–0.05, 0.14]	0.539	1.08 [0.74, 1.56]
Klotho	D0 BL vs. D8 End‐Ex	D0 BL	D8 End‐Ex	0.61 (0.26)	0.64 (0.31)	0.03 [–0.06, 0.13]	0.800	1.05 [0.72, 1.53]
Klotho	D8 BL vs. D8 End‐Ex	D8 BL	D8 End‐Ex	0.66 (0.36)	0.64 (0.31)	−0.02 [–0.11, 0.08]	0.971	0.98 [0.68, 1.40]

Biomarker concentrations are expressed in ng · mL^−1^. Time points are defined as follows: D0 BL: resting baseline before heat acclimation; D0 End‐Ex: end‐of‐exercise before heat acclimation; D8 BL: resting baseline after 7 days of heat acclimation; D8 End‐Ex: end‐of‐exercise after 7 days of heat acclimation. Mean differences represent estimated marginal mean differences between time points with 95% confidence intervals (CI). Fold change refers to the ratio of estimated marginal means (comparison/reference) with 95% CI. Statistically significant results are indicated by an asterisk (*P < 0.05; **P < 0.01; ***P < 0.001).

### 3.3 Effect of exercise on acute biomarker responses

No significant day × session interactions were detected (all *P* ≥ 0.613) for any biomarker, suggesting that the passive heat exposure protocol did not meaningfully alter the acute exercise response of these hormones. Direct comparisons of the change scores (End‐Ex‐BL) between D0 and D8 further confirmed these findings (Table 2 and Figure 1). For BDNF, the mean difference in change scores was 0.09 ng · mL^−1^ (95% CI: –1.11 to 0.94; *P* = 0.858). For irisin, the change score difference was –0.08 ng · mL^−1^ (95% CI: –0.57 to 0.41; *P* = 0.718). Klotho showed a mean difference of –0.03 ng · mL^−1^ (95% CI: –0.07 to 0.02; *P* = 0.208).

## 4 DISCUSSION

This study examined whether a seven day passive heat acclimation protocol could augment circulating levels of the biomarkers BDNF, irisin, and klotho, both at rest and in response to a standardized exercise–heat stress test. Following seven consecutive days of warm‐water immersion, resting BDNF and irisin concentrations increased by 73% and 64%, respectively, whereas klotho remained unchanged. The intervention did not meaningfully modify the acute exercise‐induced responses of any biomarker. Although baseline BDNF and irisin were elevated after acclimation, no significant transient exercise responses were observed. These findings suggest that short‐term passive heat acclimation increases resting concentrations of some biomarkers but does not modify their acute response to exercise–heat stress.

### 4.1 Effect of passive heat acclimation on resting biomarker concentrations

The observed increases in resting BDNF following passive heat acclimation are consistent with recent findings demonstrating that repeated heat exposure elevates this biomarker (Glazachev et al., 2020). This supports the growing evidence that repeated passive heat stress can activate molecular pathways involved in tissue protection, stress resilience, and neuroplasticity (McCormick et al., 2025; Patrick & Johnson, 2021). Glazachev et al. (2020) found that repeated passive heat exposure via a head‐out dry sauna (24 sessions over 10 weeks; 20–40 min at 70–80°C) significantly increased resting serum BDNF in young adults (mean age 20.2(1.6) y), compared to baseline and a thermoneutral control (25°C). This BDNF upregulation was accompanied by improved quality of life and reduced trait anxiety (a general predisposition to experience anxiety) and state anxiety (transient anxiety responses to specific situations). Further supporting these findings, a mouse model showed that 10 days of heat acclimation elevated BDNF in the medial preoptic hypothalamus, a brain region central to thermoregulation (Chen et al., 2025). This was associated with lower body core temperature and reduced anxiety during subsequent heat exposures. Together, these findings suggest that thermal stress, even in the absence of exercise, can stimulate neurotrophic signalling and may promote improvements in psychological well‐being and thermoregulatory efficiency.

Irisin showed a similar pattern of increase after passive heat acclimation in the present study. Although speculative, the mechanism may be related to heat‐induced oxidative stress and tissue signalling. Irisin release may be triggered by thermal stress as a protective response, since irisin is thought to play a role in reducing oxidative stress and cellular damage (Wang et al., 2020). Moreover, irisin and BDNF may interact as part of a muscle–brain crosstalk mechanism. Irisin can cross the blood–brain barrier and stimulate BDNF expression in the hippocampus, enhancing cognitive function and neuroprotection (Jodeiri Farshbaf & Alviña, 2021). Consequently, the simultaneous increase in both factors following heat acclimation could indicate an interconnected adaptation, potentially mediated by oxidative stress (Park et al., 2021; Wang et al., 2006), heat shock protein signalling (Franks et al., 2023; Mu et al., 2023), or neuroprotection, as discussed above.

In contrast to BDNF and irisin, circulating klotho concentrations remained stable across the seven day passive heat acclimation protocol. Klotho expression can be altered by a variety of factors under both physiological and pathological conditions, including circulatory stress, oxidative stress, hypertension, and diabetes (Prud'homme et al., 2022). To the authors' knowledge, this is the first analysis of the effects of passive heat acclimation on klotho concentrations. While acute exercise in the heat has been shown to increase circulating klotho more than exercise in temperate conditions (King et al., 2022, 2023), our findings suggest that short‐term passive heat acclimation may not sufficiently activate the renal or metabolic stress pathways required for this response. Unlike BDNF and irisin, which are linked to skeletal muscle (Paoletti & Coccurello, 2024; Rentería et al., 2022) and neuronal stress (McCormick et al., 2025; Patrick & Johnson, 2021), klotho is more closely associated with renal and endocrine function (Prud'homme et al., 2022). Without the combined stressors of exercise (e.g., haemodynamic load and fluid‐electrolyte shifts), repeated passive heat exposures alone may be inadequate to stimulate klotho upregulation. Alternatively, the stimulus applied in this study may also have been insufficient, and the thermal load may not have been large or sustained enough to elicit measurable changes in circulating klotho.

### 4.2 Effect of exercise on acute biomarker responses

A secondary aim of our study was to evaluate how acute exercise‐induced responses in BDNF, irisin, and klotho might be modulated by seven consecutive days of warm‐water immersion. Despite elevated baseline BDNF and irisin concentrations following passive heat acclimation, no significant acute exercise responses were observed, and these responses did not differ between D0 and D8, suggesting a stable acute pattern superimposed on an elevated baseline. This contrasts with findings in young adults, where 10 sessions of infrared heat exposure increased acute BDNF and irisin without elevating resting concentrations (Glazachev et al., 2021). The absence of an acute exercise response may partly relate to the timing of blood collection. Both BDNF and irisin exhibit transient post‐exercise kinetics, with reported peaks occurring anywhere from immediately to 24 h after exercise (Curtis et al., 2024; Tommasini et al., 2024). In the present study, blood was analysed from the sample collected immediately at the end of the exercise–heat stress test, which may have prevented detection of delayed or secondary peaks, potentially underestimating the overall magnitude of the acute response. The differing results may reflect age‐related attenuation of BDNF and irisin responses, potentially due to altered release dynamics (Kirby et al., 2025), and impaired downstream signalling pathways (Gooney et al., 2004) in older adults. Additionally, our participants were highly aerobically fit (mean ${\dot{V}}_{O_{2}\mathrm{peak}}$ in the ~80th age‐specific percentile) (Rapp et al., 2018), and higher fitness levels have been associated with blunted acute BDNF responses, possibly due to enhanced tissue uptake or clearance mechanisms that support long‐term adaptation (Máderová et al., 2019). Irisin exhibited a similar non‐significant post‐exercise pattern, which may reflect the balance between the substantial but well‐tolerated thermal and cardiovascular load of the protocol and the participants’ high aerobic fitness. Endurance‐trained older adults exhibit more efficient thermoregulation and reduced systemic stress during exercise (Stapleton et al., 2015), which may have limited the stimulus required to elicit a robust irisin response.

Klotho also showed no acute change in either trial, reinforcing the idea that not all protective factors respond readily to short‐term stress in older individuals. Our findings suggest that klotho is relatively stable in the face of short‐term exercise or passive heat stimuli in healthy older males, at least within the limits of our intervention. As discussed above, an upregulation of klotho may require more prolonged stress or intensive stimuli (e.g., high mechanical load or longer exposure to heat) to exhibit acute changes.

### 4.3 Limitations

Several limitations should be considered. The study involved only healthy, habitually active older men, limiting generalizability to women, sedentary individuals, athletes, or those with chronic conditions such as hypertension or type 2 diabetes. While recruitment was not specific to individuals of high aerobic fitness, the demanding nature of the study likely resulted in a self‐selection bias toward fitter participants. Nonetheless, as explained further by Janetos et al., 2025, this proof‐of‐concept design was intended to establish the feasibility and safety of the protocol under controlled laboratory conditions, providing a foundation for future trials. Sample size calculations were performed for the study's primary outcomes (Janetos et al., 2025), with analyses of biomarkers being secondary and exploratory. While 7 days of passive heat acclimation elevated BDNF and irisin, the protocol may have been too short or insufficiently intense to alter klotho concentrations. The absence of a thermoneutral or exercise‐only control group prevents full attribution of changes to heat exposure, as immersion itself or other non‐thermal factors may have contributed. Although delivered under controlled conditions, the scalability of this approach outside the laboratory is also uncertain. Future work should assess whether similar benefits can be achieved using less intensive or home‐based strategies that prioritize accessibility, adherence, and safety in heat‐vulnerable populations. Finally, the study measured circulating concentrations only, without examining tissue‐specific responses or underlying molecular mechanisms. Longitudinal studies integrating hormonal, molecular, and functional outcomes are needed to confirm these findings and clarify the role of passive heat acclimation in supporting physiological resilience and healthy ageing.

### 4.4 Conclusions

Completing seven days of warm‐water immersion significantly increased resting concentrations of BDNF and irisin in healthy, habitually active older men, suggesting that repeated passive heat exposure can enhance biomarkers linked to cognitive and metabolic health. These adaptations occurred without changes in klotho, indicating biomarker‐specific sensitivity to short‐term heat stress. Acute exercise in the heat performed before and after the intervention did not further elevate BDNF, irisin, or klotho, suggesting that repeated exposure primarily augments baseline levels rather than amplifying acute responses.Overall, these findings indicate that repeated passive heat exposure may modulate biomarkers involved in neuroplastic and metabolic regulation, supporting its potential to stimulate cellular pathways associated with physiological resilience. However, the applicability of these results to less active or clinical populations remains uncertain and warrants further investigation.

## AUTHOR CONTRIBUTIONS

Glen P Kenny designed the trial, conceived the research question and acquired funding. Kristina‐Marie T Janetos, James J McCormick and Kelli E King collected data. Joel M Garrett and James J McCormick analysed data. Joel M Garrett prepared tables and figures. Joel M Garrett drafted the manuscript. All authors revised the manuscript, approved the final version, and agree to be accountable for all aspects of the work. All persons designated as authors meet the International Committee of Medical Journal Editors (ICMJE) criteria for authorship. All authors had full access to and accept responsibility for the data presented in this report.

## CONFLICT OF INTEREST

None declared.

## Data Availability

De‐identified participant data are available from the corresponding author (gkenny@uottawa.ca) upon reasonable request and a signed access agreement.
